# Supplementary material for: BRD9 determines the cell fate of hematopoietic stem cells by regulating chromatin state
Source: Nat Commun. 2023 Dec 15;14:8372. doi: 10.1038/s41467-023-44081-6 (PMC10724271; doi:10.1038/s41467-023-44081-6)
Supplement: Supplementary file 1 — Supplementary Information [file 41467_2023_44081_MOESM1_ESM.pdf]

Supplementary Figures

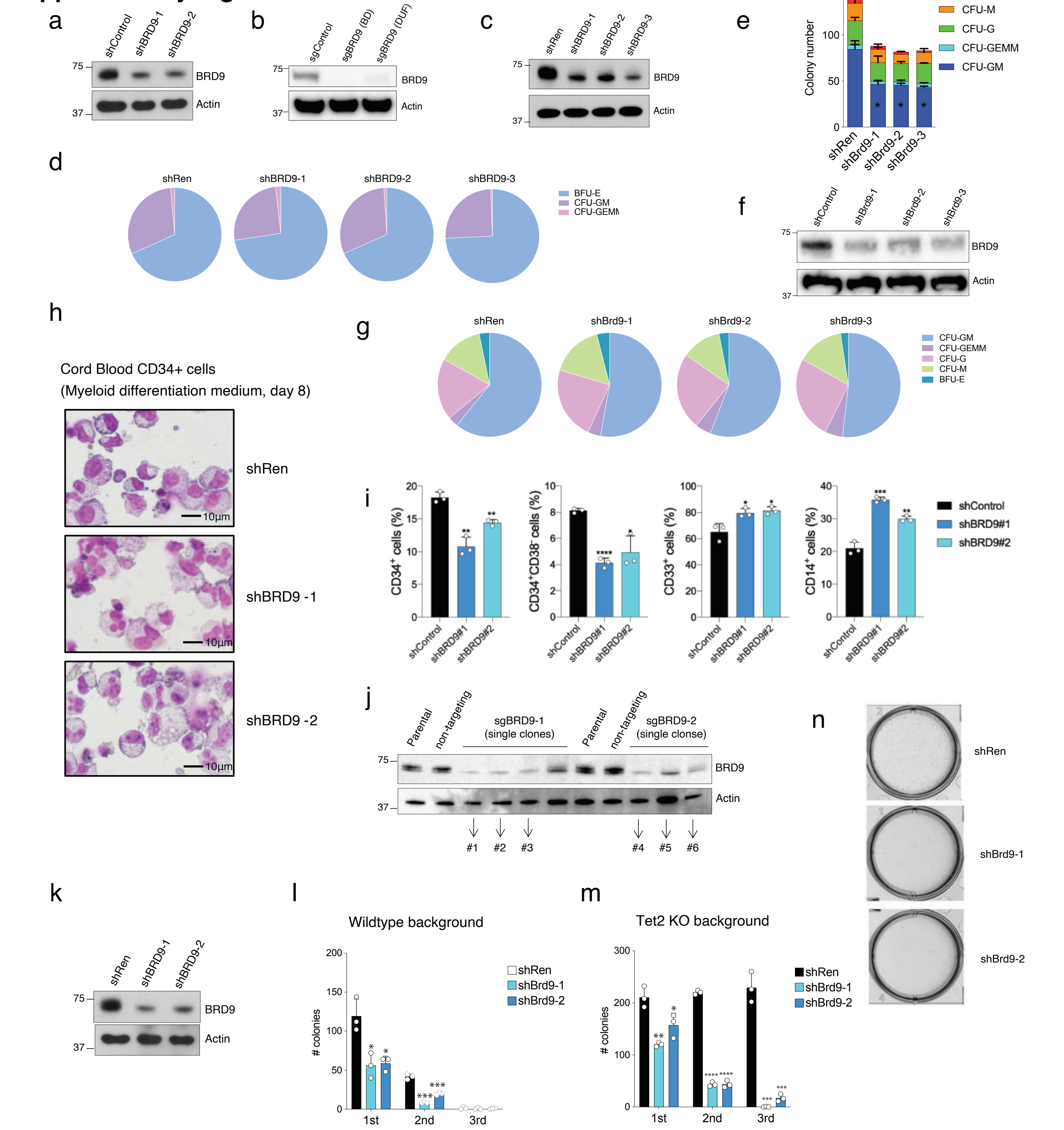

Supplementary Figure 1. BRD9 is required for normal differentiation and stemness of HSCs.
(a) Related to Fig. 1a, Western blot for BRD9 (top) and Actin (bottom). (b) Related to Fig. 1b, Western blot for BRD9 (top) and Actin (bottom). (c-d) Related to Fig. 1c, Western blot (c) for BRD9 (top) and Actin (bottom) and the proportion of BFU-E, CFU-GM, CFU-GEMM colonies (d) of human CD34+ cord blood cells transduced with a renilla (control) or BRD9-targeting shRNA. (e) Colony formation of mouse whole BM cells transduced with a renilla (control) or Brd9-targeting shRNA. Cells were sorted on the basis of GFP positivity 3 days after lentiviral transduction and colonies were scored 10 days after plating in methylcellulose. n = 2 independent experiments. (f-g) Related to (e), Western blot (f) for Brd9 (top) and Actin (bottom) and the proportion (g) of CFU-GM, CFU-GEMM, CFU-G, CFU-M and BFU-E colonies. (h) Representative cytospin image of shControl (shRen) or shBRD9-transduced human CD34+ HSCs 8 days after plating under the condition with myeloid expansion supplement. (n = 3 independent experiments). (i) Related to Fig. 1d, frequency of CD34+, CD34+CD38- (Fig. 1d, top), CD33+, and CD14+ (Fig. 1d, bottom) fractions, n = 3 independent experiments. (j) Related to Fig. 1f, Western blot with anti-BRD9 (top) and anti-Actin (bottom). Two sgRNAs and three independent clones per sgRNA were used. (k) Related to Fig. 1e, Western blot for BRD9 (top) and Actin (bottom). (l) Colony formation of murine wildtype c-Kit+ cells transduced with shRen and shBrd9. Cells were sorted on the basis of GFP positivity 3 days after lentiviral transduction and colonies were scored 10 days after plating in methylcellulose. n = 3 independent experiments. (m) As in (l), but for murine Tet2 knocked-out c-Kit+ cells obtained from Mx1-Cre;Brd9fl/fl mice treated with polyinosinic-polycytidylic acid (pIpC). n = 3 independent experiments. (n) Representative image of (l). Error bars, means ± s.e.m. *$p < 0.05$, **$p < 0.01$, ***$p < 0.001$, and ****$p < 0.0001$, two-tailed Student's t test. Source data are provided as a Source Data file.

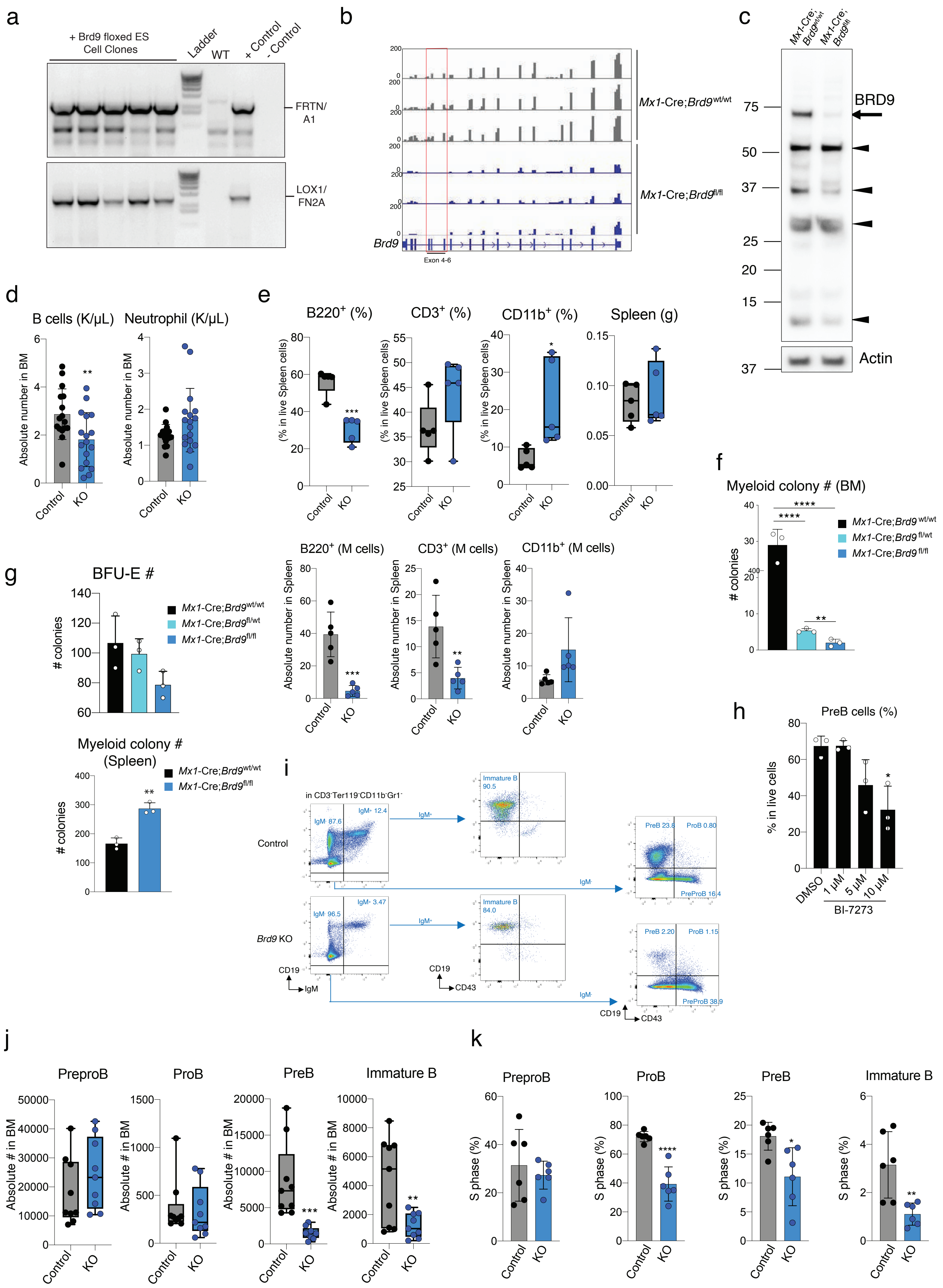

a
+ Brd9 floxed ES Cell Clones
Ladder
WT
+ Control
- Control
FRTN/ A1
LOX1/ FN2A
b
Mx1-Cre;Brd9wt/wt
Mx1-Cre;Brd9fl/fl
Brd9
Exon 4-6
c
Mx1-Cre; Brd9wt/wt
Mx1-Cre; Brd9fl/fl
BRD9
Actin
75
50
37
25
20
15
37
d
B cells (K/μL)
Neutrophil (K/μL)
Absolute number in BM
Control
KO
e
B220+ (%)
CD3+ (%)
CD11b+ (%)
Spleen (g)
(% in live Spleen cells)
B220+ (M cells)
CD3+ (M cells)
CD11b+ (M cells)
Absolute number in Spleen
f
Myeloid colony # (BM)
colonies
Mx1-Cre;Brd9 wt/wt
Mx1-Cre;Brd9 fl/wt
Mx1-Cre;Brd9 fl/fl
g
BFU-E #
colonies
Mx1-Cre;Brd9wt/wt
Mx1-Cre;Brd9fl/wt
Mx1-Cre;Brd9fl/fl
Myeloid colony # (Spleen)
Mx1-Cre;Brd9wt/wt
Mx1-Cre;Brd9fl/fl
h
PreB cells (%)
% in live cells
DMSO
1 μM
5 μM
10 μM
BI-7273
i
in CD3-Ter119-CD11b-Gr1-
Control
Brd9 KO
IgM- 87.6
IgM+ 12.4
IgM- 96.5
IgM+ 3.47
IgM+
IgM-
Immature B 90.5
Immature B 84.0
PreB 23.8
ProB 0.80
PreProB 16.4
PreB 2.20
ProB 1.15
PreProB 38.9
CD19
IgM
CD43
j
PreproB
ProB
PreB
Immature B
Absolute # in BM
k
PreproB
ProB
PreB
Immature B
S phase (%)
Control
KO

Supplementary Figure 2. Phenotypic analysis of the hematopoietic system in Brd9 knockout murine model. (a) Confirmation of expanded clones (top) and of LoxP retention by PCR using primers A1 and FRTN (top) and LOX1 and FN2A (bottom) shown in Fig. 2a. All five clones are Brd9 floxed ES cell clones. (b) Genomic track of RNA-seq results shows the deletion of exons of Brd9. RNA-seq reads on exon 4 to 6 (red rectangle) show a significant decrease upon pIpC-induced deletion. (c) Related to Fig. 2c, Western blot with anti-BRD9 (top) and anti-Actin (bottom). BRD9 and nonspecific bands are indicated by the arrow and triangles, respectively. (d) Related to Fig. 2d, absolute number of B cells and Neutrophils in the BM cells of each group. n = 16 (Control) and n = 17 (KO) in B cells and Neutrophils, both males and females. (e) Frequency of B220+, CD3+, and CD11b+ cells in spleen of 4-month-old Mx1-Cre;Brd9WT/WT (Control) mice and Mx1-Cre;Brd9fl/fl mice cKO (KO) mice. Spleen weight is also shown in the right. Absolute number of B220+, CD3+, and CD11b+ cells in spleen was indicated at the bottom. n = 5 independent experiments, males. (f) Related to Fig. 2e, the results of independent colony formation assay of whole BM cells were shown. Colonies were scored 10 days after 4th plating in methylcellulose M3434 for myeloid colonies. n = 3 independent experiments. (g) Colony formation of whole BM cells and spleen cells. Colonies were scored 10 days after plating in methylcellulose M3436 and M3434 for BFU-E colonies (top) and myeloid colonies (bottom), respectively. n = 3 independent experiments. (h) Related to Fig. 2f, frequency of Pre-B cells in live colony cells was evaluated by FACS analysis. n = 3 independent experiments. (i) FACS analysis and gating strategy of BM cells from representative primary mice to evaluate B cell development. (j) Related to Fig. 2g, Box-and-whisker plots of numbers of PreproB, ProB, PreB and Immature B. n=9 independent samples, both males and females. (k) Bromodeoxyuridine (BrdU)+ (S-phase) proportion of each B cell fraction in the BM of primary 12-week-old Brd9 KO and control mice. n = 6 independent experiments, males. Error bars, means ± s.e.m. *$p < 0.05$, **$p < 0.01$, ***$p < 0.001$, and ****$p < 0.0001$, two-tailed Student’s t test. Source data are provided as a Source Data file.

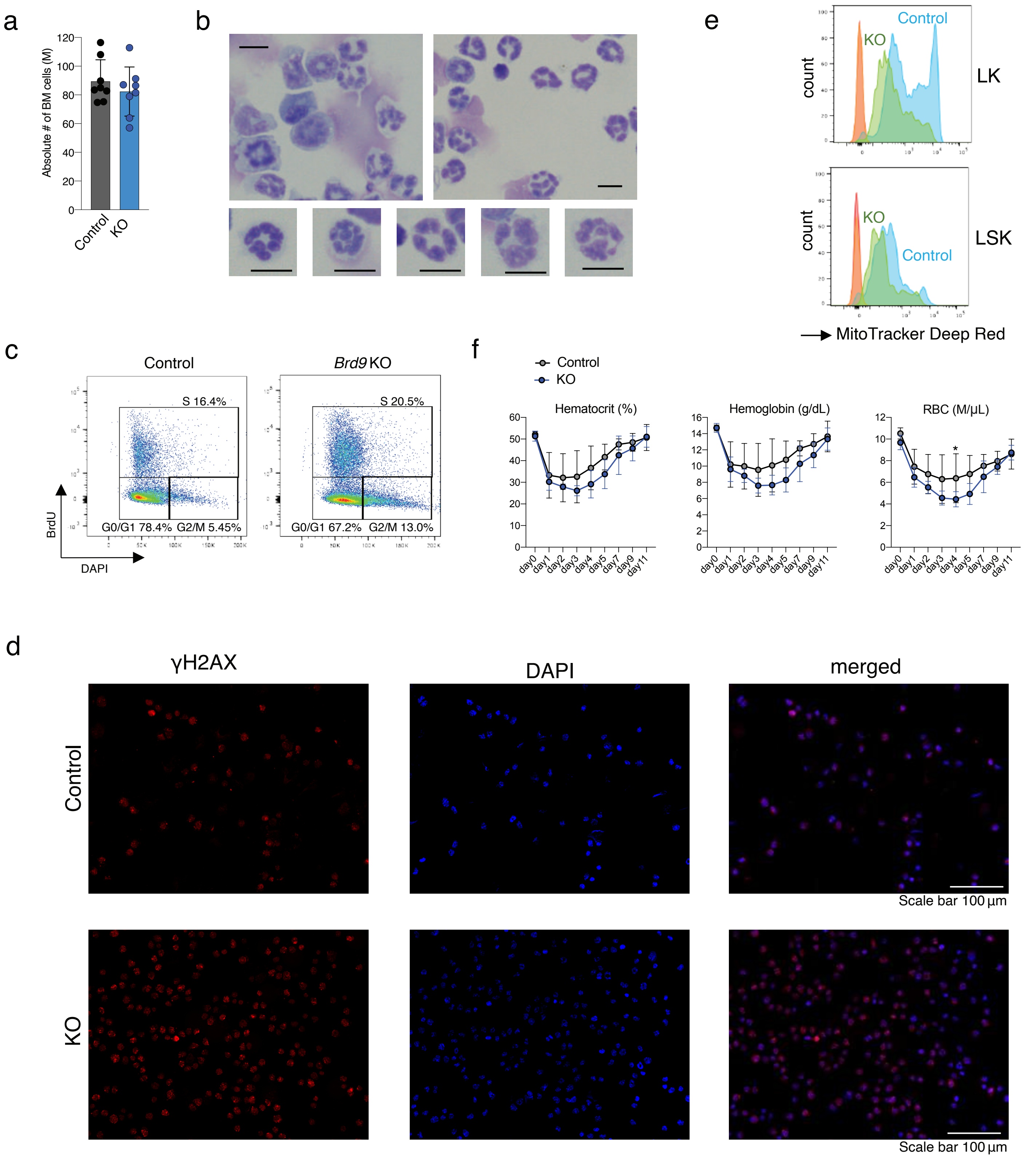

Supplementary Figure 3. Phenotypic analysis of Brd9 knockout hematopoietic cells.
(a) Numbers of live mononuclear BM cells. The absolute number contained in femurs, tibias, and pelvis is shown. n = 8 independent experiments. (b) BM cytospins indicating aberrantly segmented, hypogranular myeloid cells in KO BM. Bar: 10 μm. (c) Representative FACS plots of LSK cells to evaluate cell cycle. (d) Related to Fig. 2h, representative images of gH2AX (red) immunofluorescence staining and counterstaining with DAPI (blue) in HSPCs. (e) Representative FACS plots of mitochondria membrane potential (MMP) in LK (top) and LSK (bottom) cells, as measured by MitoTracker Deep Red. blue, Control; green, KO; Unstained control, red. (f) Analysis of hematocrit, hemoglobin, and RBC during recovery from the treatment with PHZ (day 0, 50 mg/kg). Control, n=6 independent samples;KO, n=8 independent samples. Error bars, means ± s.e.m. *$p < 0.05$, two-tailed Student’s t-test. Source data are provided as a Source Data file.

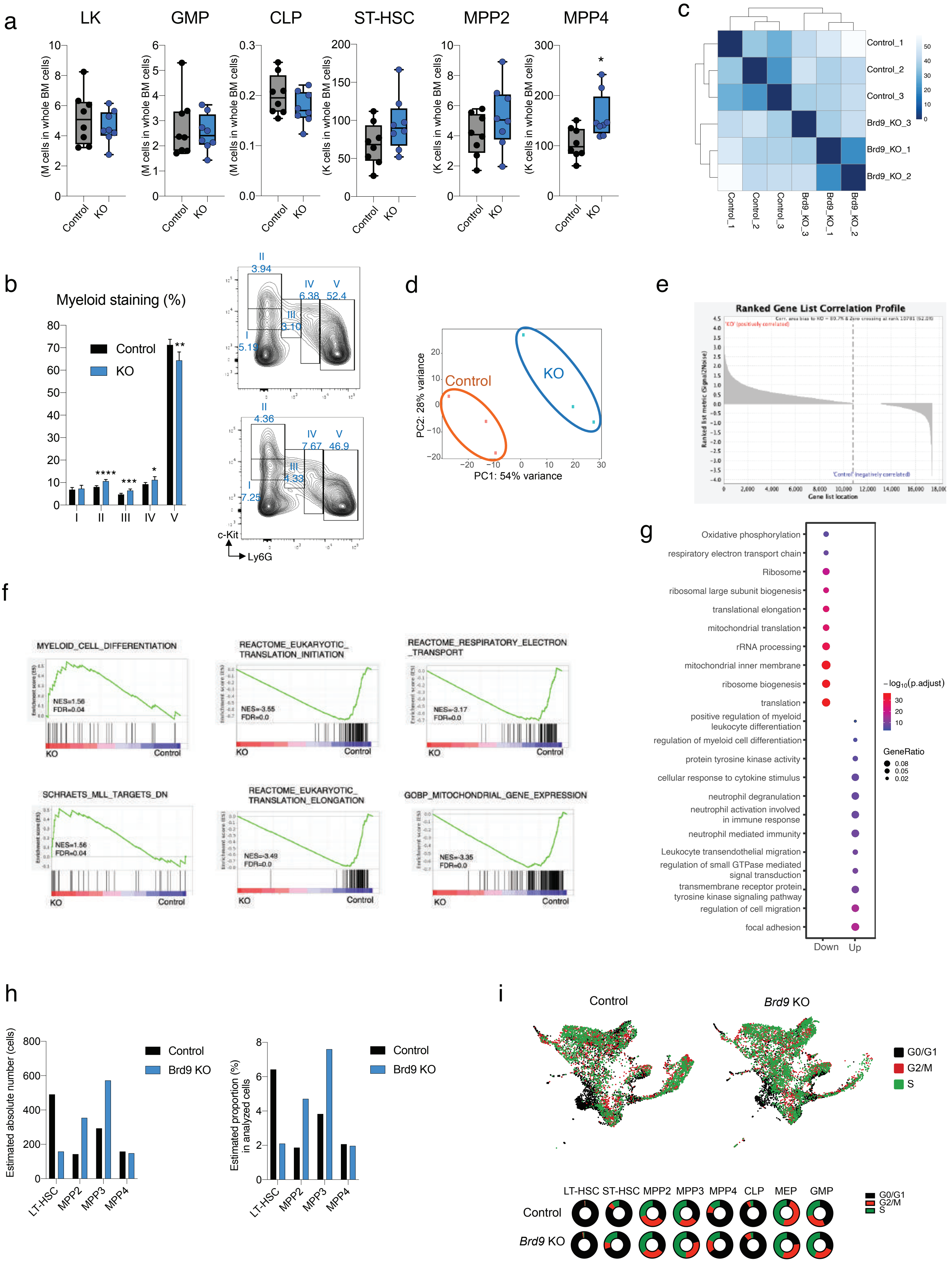
a
LK
GMP
CLP
ST-HSC
MPP2
MPP4
(M cells in whole BM cells)
(K cells in whole BM cells)
Control
KO
c
Control_1
Control_2
Control_3
Brd9_KO_3
Brd9_KO_1
Brd9_KO_2
b
Myeloid staining (%)
Control
KO
I
II
III
IV
V
c-Kit
Ly6G
d
Control
KO
PC1: 54% variance
PC2: 28% variance
e
Ranked Gene List Correlation Profile
Gene list location
f
MYELOID_CELL_DIFFERENTIATION
NES=1.56
FDR=0.04
REACTOME_EUKARYOTIC_TRANSLATION_INITIATION
NES=-3.55
FDR=0.0
REACTOME_RESPIRATORY_ELECTRON_TRANSPORT
NES=-3.17
FDR=0.0
SCHRAETS_MLL_TARGETS_DN
NES=1.56
FDR=0.04
REACTOME_EUKARYOTIC_TRANSLATION_ELONGATION
NES=-3.49
FDR=0.0
GOBP_MITOCHONDRIAL_GENE_EXPRESSION
NES=-3.35
FDR=0.0
g
Oxidative phosphorylation
respiratory electron transport chain
Ribosome
ribosomal large subunit biogenesis
translational elongation
mitochondrial translation
rRNA processing
mitochondrial inner membrane
ribosome biogenesis
translation
positive regulation of myeloid leukocyte differentiation
regulation of myeloid cell differentiation
protein tyrosine kinase activity
cellular response to cytokine stimulus
neutrophil degranulation
neutrophil activation involved in immune response
neutrophil mediated immunity
Leukocyte transendothelial migration
regulation of small GTPase mediated signal transduction
transmembrane receptor protein tyrosine kinase signaling pathway
regulation of cell migration
focal adhesion
Down
Up
−log10(p.adjust)
GeneRatio
h
Estimated absolute number (cells)
Estimated proportion (%) in analyzed cells
Control
Brd9 KO
LT-HSC
MPP2
MPP3
MPP4
i
Control
Brd9 KO
G0/G1
G2/M
S
LT-HSC ST-HSC MPP2 MPP3 MPP4 CLP MEP GMP

Supplementary Figure 4. The effects of BRD9 loss on HSPCs.
(a) Box-and-whisker plots of numbers of LK, GMPs, CLPs, ST-HSCs, MPP2s, and MPP4s in BM of primary 6-month-old Brd9fl/fl (Control) and Mx1-Cre;Brd9fl/fl (KO). Bars indicate medians, box edges indicate first and third quartile values, and whisker edges indicate minimum and maximum values. n=8 independent experiments. *p < 0.05, **p < 0.01, ***p < 0.001, and ****p < 0.0001, P value relative to control by a two-sided t-test. (b) Flow cytometric analysis of CD19−CD3−CD4−CD8−Ter119−CD115− BM cells of control and KO mice (n = 5 per each group). The frequency of each fraction in nonlymphoid/nonerythroid/nonmonocytic BM cells is shown (right). Error bars, means ± s.e.m. *p < 0.05, **p < 0.01, ***p < 0.001, and ****p < 0.0001, two-tailed Student's t test. (c) Similarity matrix and hierarchical clustering of two groups (Brd9fl/fl and Mx1-Cre;Brd9fl/fl). (d) Principal component (PC) analysis of gene expression from Control and KO group, biologically triplicated in each group. (e) Ranked gene list correlation profile showing positively (left) and negatively (right) correlated in KO. (f) GSEA enrichment plot for dysregulated genes in RNA-seq of Brd9 KO vs. Control. (g) Significantly dysregulated pathways. Gene ratio and statistical significance (-log10(Adjusted P-value)) were shown. (h) Related to Fig. 3e, estimated absolute number and proportion of LT-HSC, MPP2, MPP3, MPP4 in Control and KO Lin-cKit+ BM cells. (i) Segregation of dormant and dividing HSPC population by cell cycle phase gene expression, where cell cycles were estimated by CellCycleScoring function with cc.genes.updated.2019 in Seurat. UMAP plots were made using the whole transcriptional expression data. Source data are provided as a Source Data file.

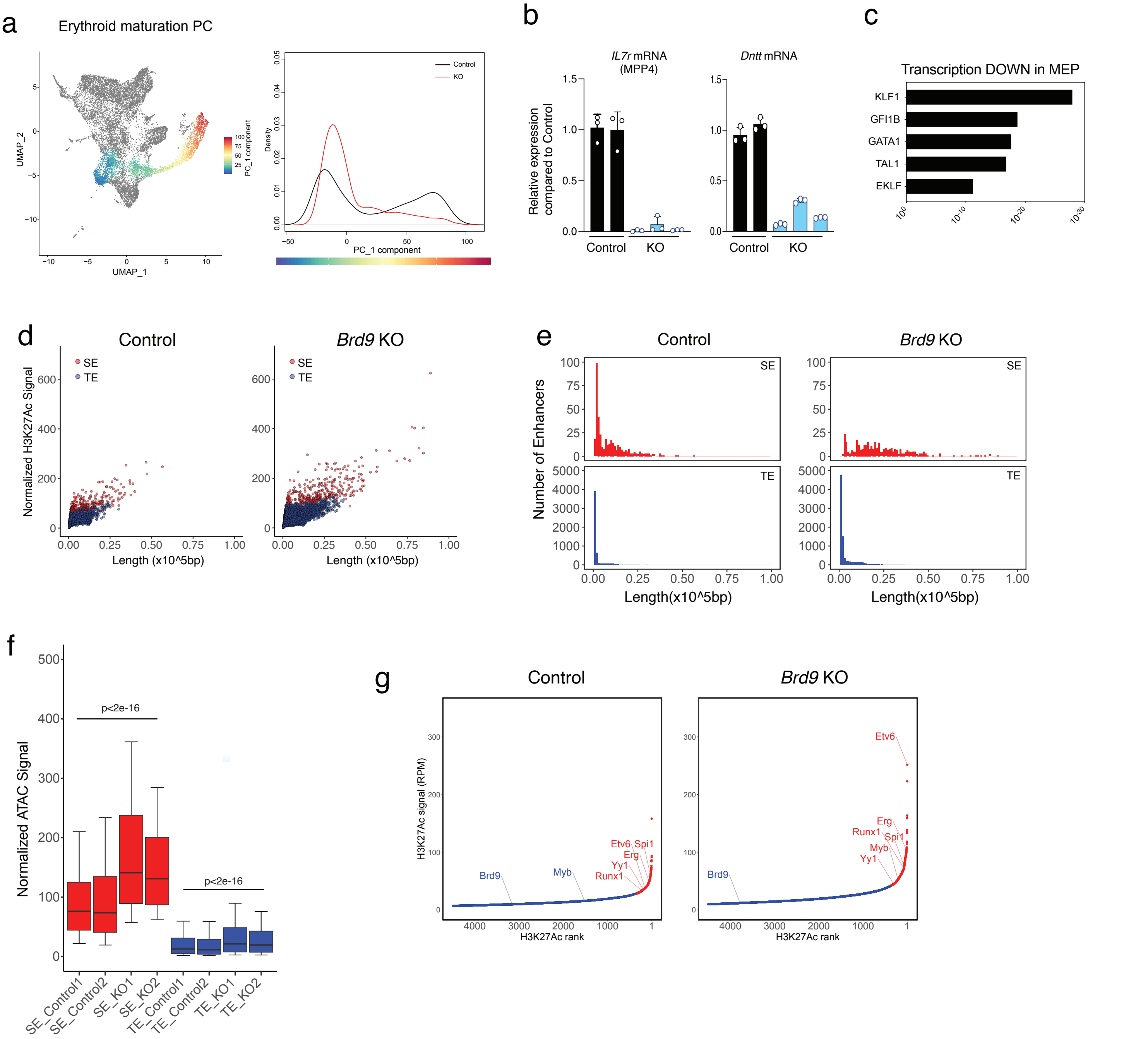

Supplementary Figure 5. BRD9 affected HSC development by regulating chromatin structure.
(a) Cellular densities along erythroid maturation PC and the scaled values of the maturation PC visualized on UMAP space. (b) Related to Fig. 3g, the expression level of Il7r and Dntt in FACS-sorted MPP4 cells of Control and KO mice was evaluated by manual qRT–PCR. n = 3 independent experiments. (c) Transcriptional factor motif analysis of genes downregulated in MEP of KO mice. P values are indicated and generated via Enricher. (d) Scatterplots of the H3K27ac signal and peak length. Red and blue indicate SEs and TEs, respectively. (e) Distribution of peak lengths for SEs (red, top) and TEs (blue, bottom). (f) ATAC-seq signal at SE, and TE (detected with HOMER fdr < 0.001) in HSPCs. Normalized signal indicates were calculated from RPGC values. Two biologically independent samples each group were used (control vs. KO). The p values were obtained by analysis of variance. In the box-and-whisker plots, the 10th, 25th, 50th, 75th and 90th percentiles are shown. (g) Same as Fig. 3i, except that mapped sequencing reads (BAM files) from two biologically independent samples, one of which was used in Fig. 3i, were combined and analyzed here. Source data are provided as a Source Data file.

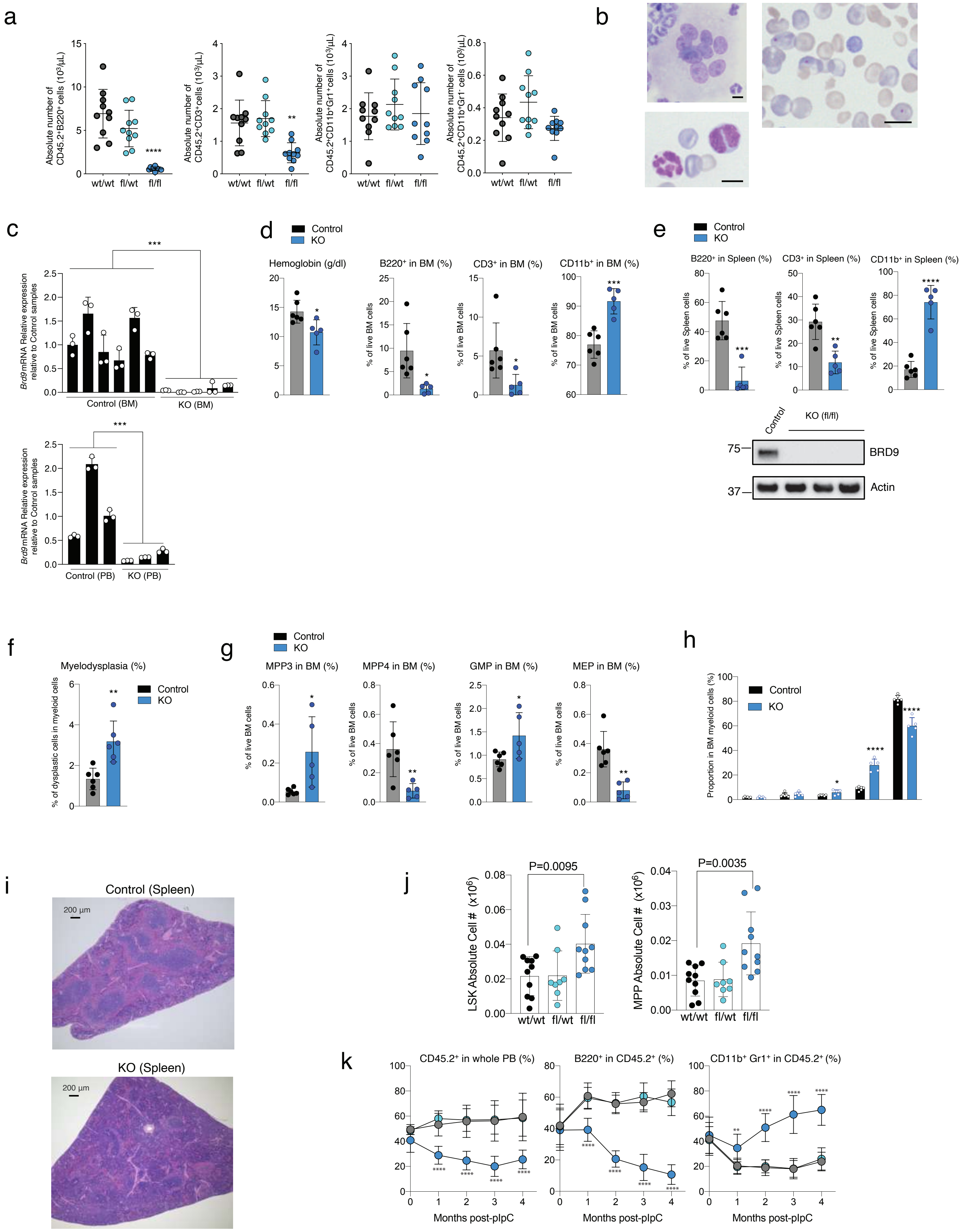
a
Absolute number of CD45.2⁺B220⁺ cells (10³/μL)
Absolute number of CD45.2⁺CD3⁺cells (10³/μL)
Absolute number of CD45.2⁺CD11b⁺Gr1⁺cells (10³/μL)
Absolute number of CD45.2⁺CD11b⁺Gr1⁻ cells (10³/μL)
wt/wt fl/wt fl/fl
****
**
b
c
***
Brd9 mRNA Relative expression relative to Cotnrol samples
Control (BM)
KO (BM)
Control (PB)
KO (PB)
d
Control
KO
Hemoglobin (g/dl)
B220⁺ in BM (%)
CD3⁺ in BM (%)
CD11b⁺ in BM (%)
% of live BM cells
e
B220⁺ in Spleen (%)
CD3⁺ in Spleen (%)
CD11b⁺ in Spleen (%)
% of live Spleen cells
Control
KO (fl/fl)
75
37
BRD9
Actin
f
Myelodysplasia (%)
% of dysplastic cells in myeloid cells
g
MPP3 in BM (%)
MPP4 in BM (%)
GMP in BM (%)
MEP in BM (%)
h
Proportion in BM myeloid cells (%)
i
Control (Spleen)
KO (Spleen)
200 μm
j
P=0.0095
P=0.0035
LSK Absolute Cell # (x10⁶)
MPP Absolute Cell # (x10⁶)
k
CD45.2⁺ in whole PB (%)
B220⁺ in CD45.2⁺ (%)
CD11b⁺ Gr1⁺ in CD45.2⁺ (%)
Months post-pIpC

Supplementary Figure 6. Cell-autonomous effects of BRD9 loss in HSPCs.

(a) Related to Fig. 4c, absolute number of CD45.2+ donor-derived B220+ cells, CD3+ cells, CD11b+Gr1+ cells, and CD11b+Gr1- cells in each group. n=10 independent experiments; mean and s.e.m are plotted. P value relative to control by a two-sided t-test. *P<0.05, **P<0.01, ***P<0.001, and ****P<0.0001. (b) Related to Fig. 4d, dysplastic features such as megakaryocyte with multiple and separated nuclei, Howell-Jolly bodies, and hyper-/hypo-segmented myeloid cells. Bar: 10 μm. (c) Related to Fig. 4d, Brd9 mRNA level in the BM (top) and representative Brd9 mRNA level in the PB (bottom) of Control and KO mice, evaluated by manual qRT-PCR. P value relative to control by a two-sided t-test. n = 3 each. (d) Plots of hemoglobin and the proportion of B220+, CD3+, and CD11b+ cells in the BM of recipient mice in (c). Mean ± SD; P value relative to control by a two-sided t-test. Control, n=6 independent samples; KO, n= 5 independent samples. (e) Plots of hemoglobin and the proportion of B220+, CD3+, and CD11b+ cells in the spleen (top) of recipient mice in (c). Mean ± SD; P value relative to control by a two-sided t-test. Control, n=6 independent samples; KO, n= 5 independent samples. Representative images of Western blot (bottom) for BRD9 in spleen cells derived from pIpCed Mx1-Cre;Brd9WT/WT (Control) mice and Mx1-Cre;Brd9fl/fl mice cKO (fl/fl) mice (n=3 independent experiments). (f) Related to Fig. 4d, frequency of myeloid dysplasia in myeloid BM cells (n = 6 per each group). Mean ± SD; P value relative to control by a two-sided t-test. (g) Proportion of MPP3, MPP4, GMPs and MEPs in the BM of Control and KO mice in (c). Mean ± SD; P value relative to control by a two-sided t-test. (h) Flow cytometric analysis of CD19−CD3−CD4−CD8−Ter119−CD115− BM cells of control and KO mice in (c). Mean ± SD; P value relative to control by a two-sided t-test. (i) Representative histology images of the spleen in (c), Bar: 200 μm. (j) Related to Fig. 4f, absolute number of donor-derived CD45.2+ LSK and MPP cells in each group. n=10 independent samples in Mx1-cre control and Mx1-cre;Brd9fl/fl group, n=8 independent samples in Mx1-cre;Brd9fl/wt group. Mean ± SD; P value relative to control by a two-sided t-test. (k) Related to Fig. 4g, percentage of CD45.2+ in the whole PB cells (left) and of B220+ (middle) and CD11b+Gr1+ cells in donor-derived CD45.2+ in primary competitive transplantation. Mean ± SD. P value relative to the control group at four months by a two-sided t-test. n=10 independent samples in Mx1-cre control and Mx1-cre;Brd9fl/fl group, n=8 independent samples in Mx1-cre;Brd9fl/wt group. *p < 0.05, **p < 0.01, ***p < 0.001, and ****p < 0.0001. Source data are provided as a Source Data file.

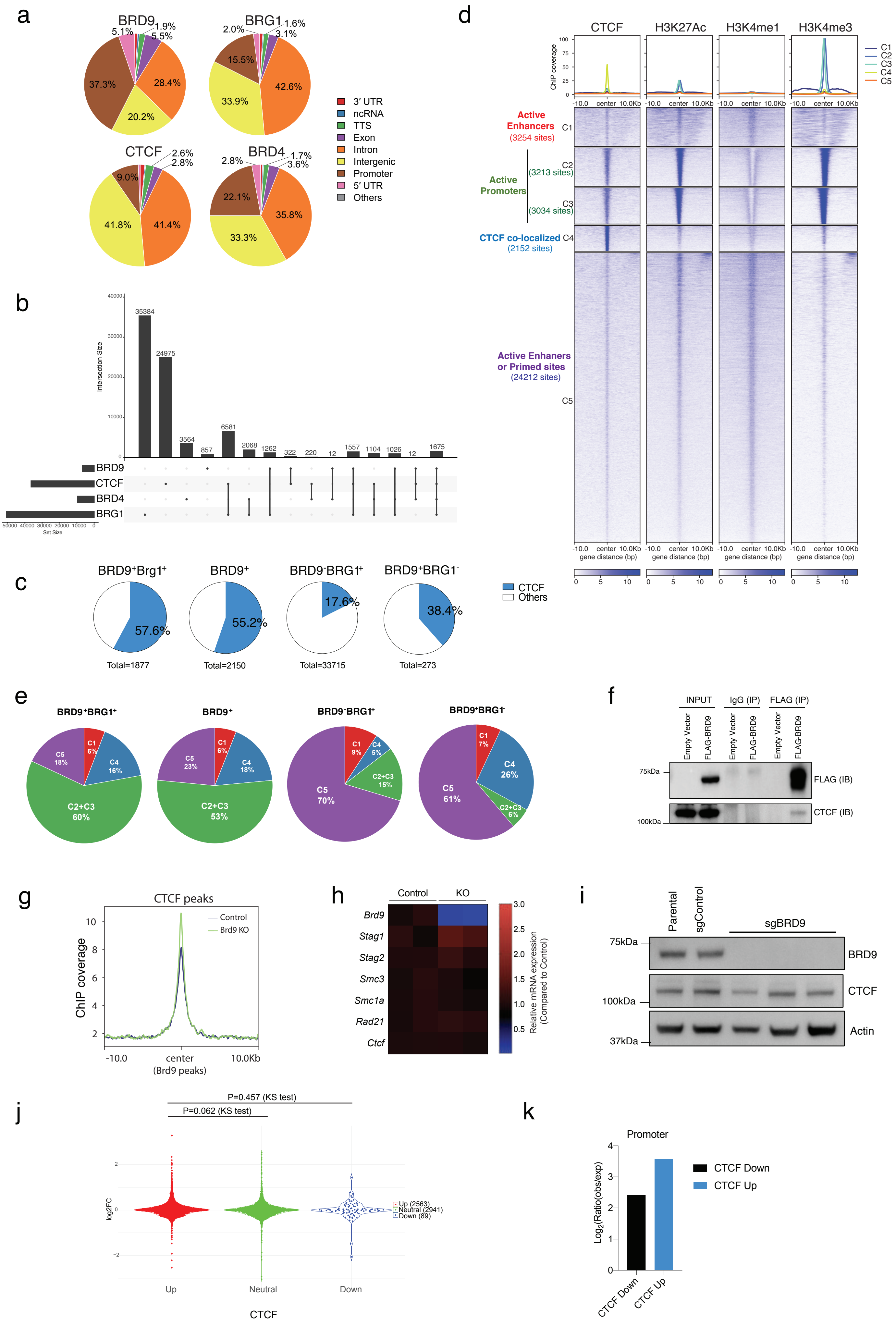

a
BRD9
1.9%
5.1%
5.5%
37.3%
28.4%
20.2%
BRG1
1.6%
2.0%
3.1%
15.5%
33.9%
42.6%
CTCF
2.6%
2.8%
9.0%
41.8%
41.4%
BRD4
1.7%
2.8%
3.6%
22.1%
35.8%
33.3%
3' UTR
ncRNA
TTS
Exon
Intron
Intergenic
Promoter
5' UTR
Others
b
Intersection Size
35384
24975
3564
857
6581
2068
1262
322
220
12
1557
1104
1026
12
1675
BRD9
CTCF
BRD4
BRG1
Set Size
c
BRD9+Brg1+
57.6%
Total=1877
BRD9+
55.2%
Total=2150
BRD9-BRG1+
17.6%
Total=33715
BRD9+BRG1-
38.4%
Total=273
CTCF
Others
d
CTCF
H3K27Ac
H3K4me1
H3K4me3
ChIP coverage
C1
C2
C3
C4
C5
Active Enhancers
(3254 sites)
Active Promoters
(3213 sites)
(3034 sites)
CTCF co-localized
(2152 sites)
Active Enhaners or Primed sites
(24212 sites)
-10.0
center
10.0Kb
gene distance (bp)
e
BRD9+BRG1+
C1 6%
C4 16%
C5 18%
C2+C3 60%
BRD9+
C1 6%
C4 18%
C5 23%
C2+C3 53%
BRD9-BRG1+
C1 9%
C4 5%
C2+C3 15%
C5 70%
BRD9+BRG1-
C1 7%
C4 26%
C2+C3 6%
C5 61%
f
INPUT
IgG (IP)
FLAG (IP)
Empty Vector
FLAG-BRD9
75kDa
100kDa
FLAG (IB)
CTCF (IB)
g
CTCF peaks
Control
Brd9 KO
ChIP coverage
-10.0
center
10.0Kb
(Brd9 peaks)
h
Control
KO
Brd9
Stag1
Stag2
Smc3
Smc1a
Rad21
Ctcf
Relative mRNA expression (Compared to Control)
i
Parental
sgControl
sgBRD9
75kDa
100kDa
37kDa
BRD9
CTCF
Actin
j
P=0.457 (KS test)
P=0.062 (KS test)
log2FC
Up (2563)
Neutral (2941)
Down (89)
Up
Neutral
Down
CTCF
k
Promoter
Log2(Ratio(obs/exp))
CTCF Down
CTCF Up

Supplementary Figure 7. Genomic distribution of BRD9 in relation to CTCF.
(a) The genomic distribution of BRD9, BRG1, CTCF, and BRD4 peaks. (b) Upset plots depicting the overlap of BRD9, BRG1, CTCF, and BRD4 in the Control condition. (c) Pie chart of BRD9+BRG1+ (ncBAF), BRD9+ (ncBAF + ncBAF-independent BRD9 targets), BRD9-BRG1+ (BAFs other than ncBAF) and BRD9+BRG1- (ncBAF-independent BRD9 targets) peaks overlapping with CTCF peaks. (d) Related to Fig. 5b, genome-wide distribution of CTCF, H3K27ac, H3K4me1, and H3K4me3 marks in HSPCs at all BAF (BRD9+ or BRG1+) peaks, clustered into five groups using K-means clustering method. (e) The proportion of BRD9+BRG1+(ncBAF), BRD9+ (ncBAF + ncBAF-independent BRD9 targets), BRD9-BRG1+ (BAFs other than ncBAF) and BRD9+BRG1- (ncBAF-independent BRD9 targets) specific peaks overlapping with specified chromatin features as in (d). (f) Immunoblots of IgG or FLAG immunoprecipitation (IP) from total cell lysate of FLAG-BRD9 cDNA transduced 293T. FLAG and CTCF antibodies were used for the blotting. (g) Coverage of ChIP-seq signal at Brd9 peaks regions. Independent data set from Fig.5d. (h) The quantitative mRNA expression of Brd9, ohesion genes (Stag1, Stag2, Smc3, Smc1a, and Rad21), and Ctcf in control and Brd9 KO conditions. (i) Immunoblots of BRD9, CTCF, and Actin in BRD9-depleted K562 single clones transduced with Cas9 and sgBRD9. (j) Related to Fig. 5g, the violin plot figure to show a distribution of altered gene expression for each category (CTCF Up, Neutral, and Down, two-sided KS test). One biologically independent sample in each condition (control vs. KO) was used. (k) Log2 ratio of the observed proportion of the CTCF peaks against the expected ones in promoter. Source data are provided as a Source Data file.

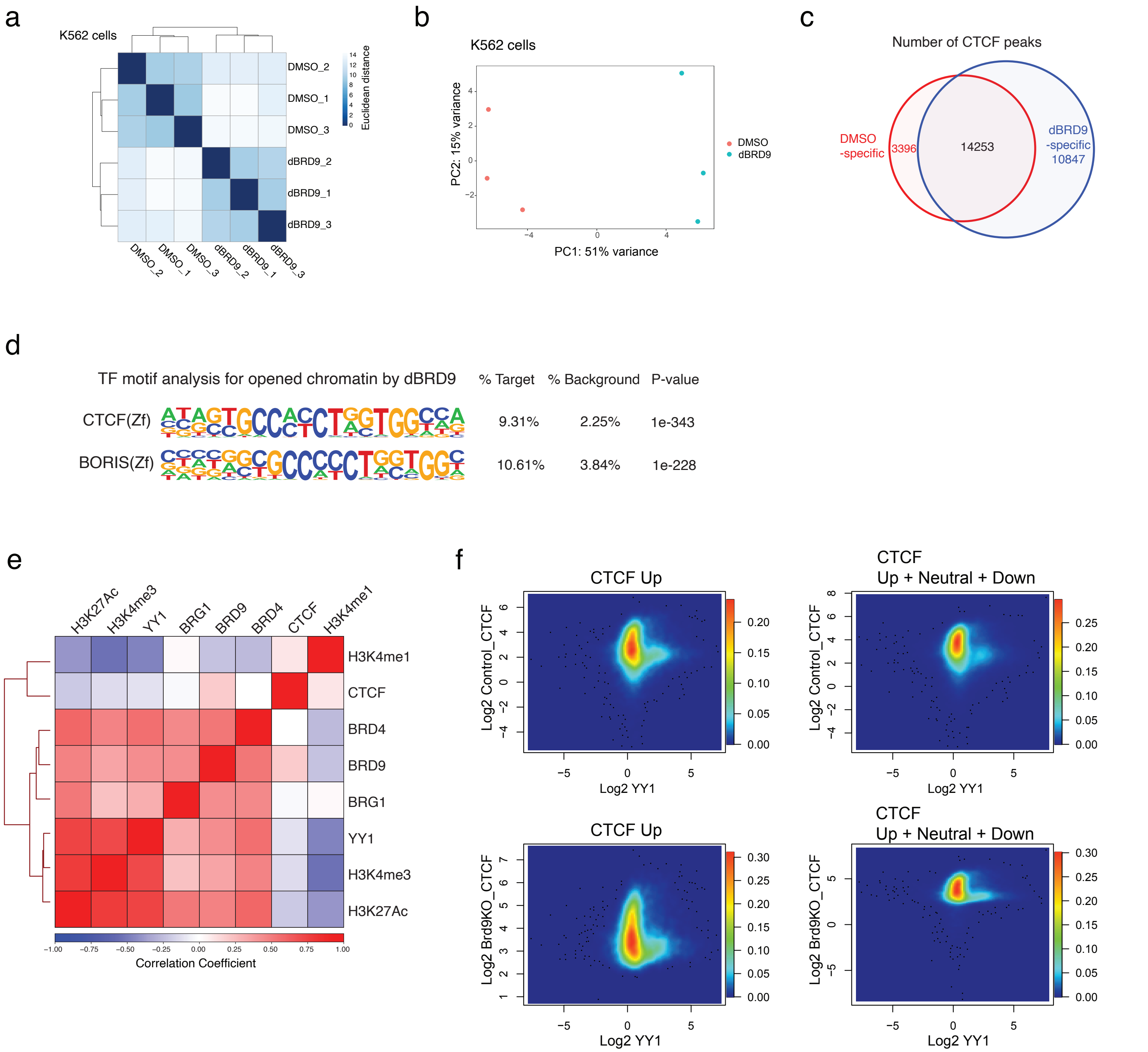

Supplementary Figure 8. BRD9 is functionally associated with CTCF and YY1.
(a) Similarity matrix and hierarchical clustering of two groups, K562 treated with DMSO or 50 nM dBRD9 for 4 days, biologically triplicated in each group. (b) Principal component (PC) analysis of gene expression. (c) Venn diagrams showing the alteration of CTCF peaks evaluated by ChIP-seq of K562 cells with DMSO or dBRD9 group.
(d) Transcriptional factor motif analysis for opened chromatin in K562 cells treated with dBRD9. (e) Heatmap representing the correlations between average ChIP-seq reads (log2[reads per genome coverage (RPGC)]) over a merged set of BRD9, BRG1, CTCF, BRD4, YY1, H3K4me1, H3K4me3, and H3K27ac peaks. (f) Density plots of YY1 and CTCF ChIP signals at CTCF peaks. Two vertical panels on the left-hand side and on the right-hand side indicate densities at the CTCF Up peaks in Brd9 KO condition and at the all CTCF peaks, respectively. Source data are provided as a Source Data file.

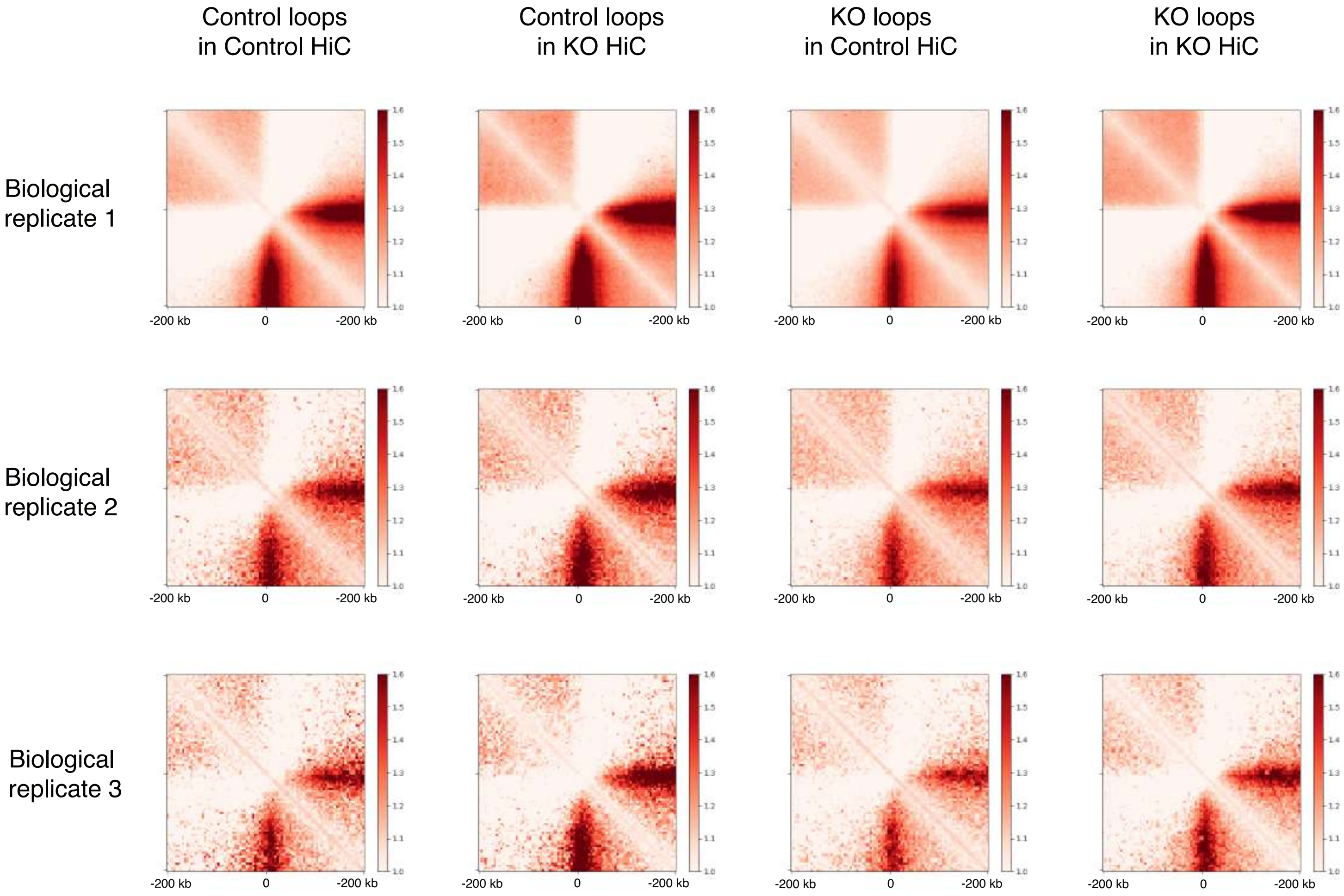

Supplementary Figure 9. Distinction of loop intensities aggregated over the Control and KO loops detected in the first biological replicate was maintained in the second and third biological replicates. We carried out the aggregate analysis of loop intensities over the 2199 Control and 4640 KO loops detected in the first biological replicate to Control and KO HiC maps of the three biological replicates. The zero coordinate on x axis represents 5' anchors of the loops, and the loops extend in the positive direction on x axis. The negative x coordinate (-200 kb to 0) represents the genomic regions upstream of the loops, thus showing the profile of background loop intensities over non-loop regions. The value (color) of each pixel (5 kb resolution) represents the average of loop intensities aggregated in the pixel. For the second and third biological replicates, we created contact frequency maps (hic files). The HiC aggregate analyses were performed by using FAN-C (Kruse et al. 2019).

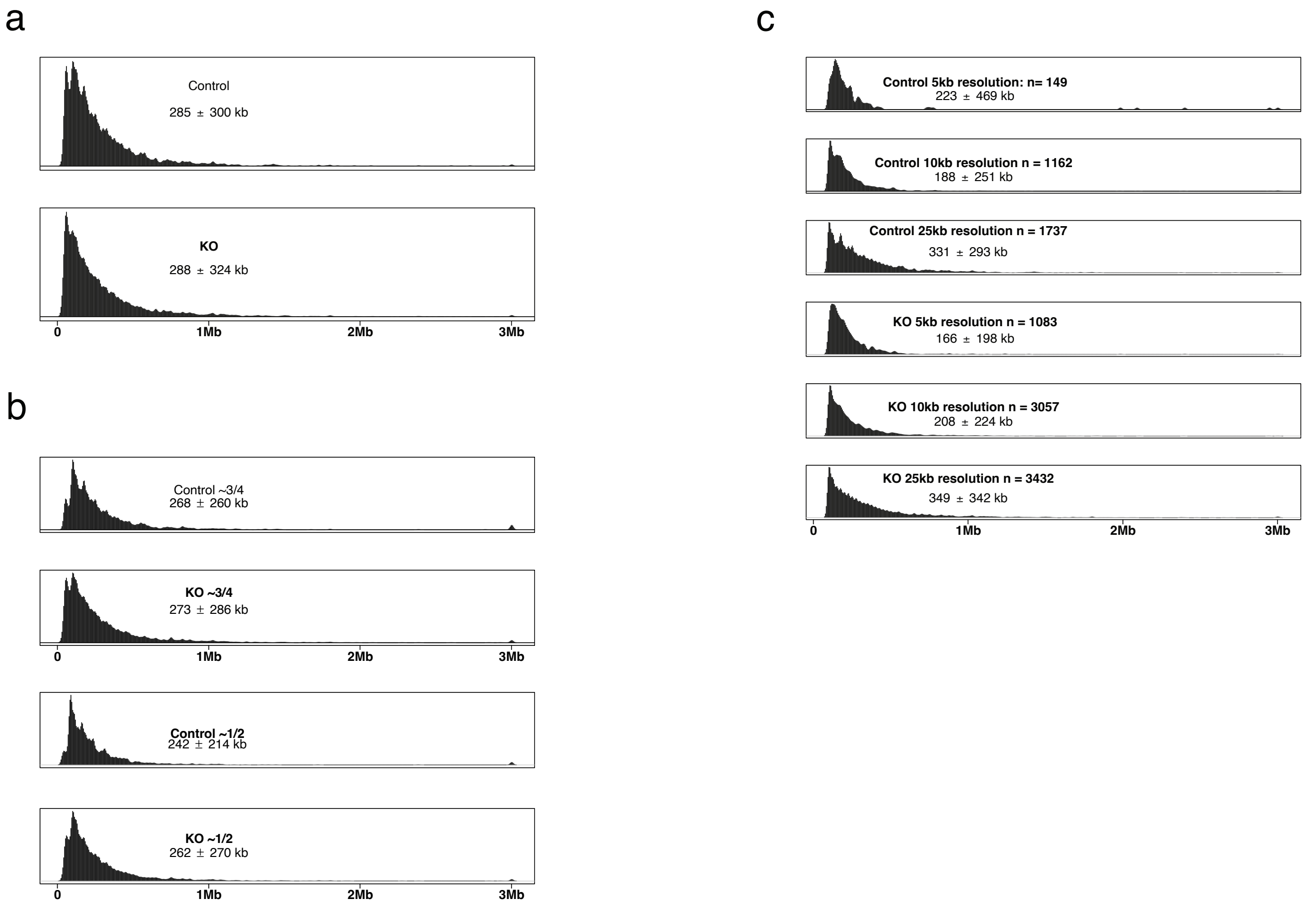

Supplementary Figure 10. Numbers and sizes of chromatin loops detected at distinct mapped sequence depth and at distinct resolutions.
The distribution of loop sizes is shown for loops detected in each of the whole merged datasets of Control and KO cells (a), datasets of decreased numbers of read-pairs (b), and for loops detected at the three resolutions, e.g., 5,10 and 25 kb (c). The number of loops and average and sd. of the loop sizes are given in each panel.

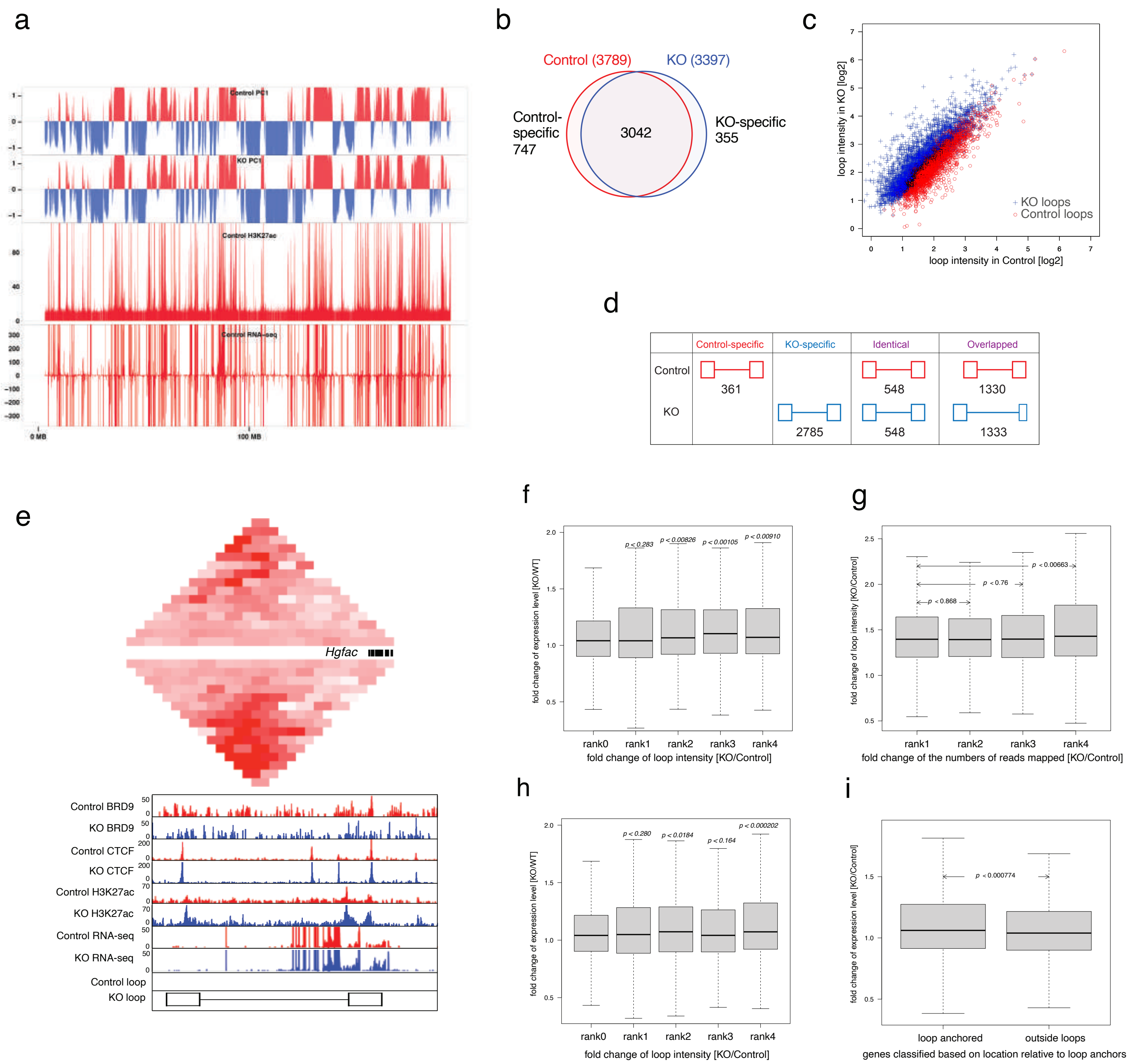
a
Control PC1
KO PC1
Control H3K27ac
Control RNA-seq
0 MB
100 MB
b
Control (3789)
KO (3397)
Control-specific 747
3042
KO-specific 355
c
loop intensity in KO [log2]
loop intensity in Control [log2]
KO loops
Control loops
d
Control-specific
KO-specific
Identical
Overlapped
Control
KO
361
2785
548
548
1330
1333
e
Hgfac
Control BRD9
KO BRD9
Control CTCF
KO CTCF
Control H3K27ac
KO H3K27ac
Control RNA-seq
KO RNA-seq
Control loop
KO loop
f
fold change of expression level [KO/WT]
p < 0.283
p < 0.00826
p < 0.00105
p < 0.00910
rank0
rank1
rank2
rank3
rank4
fold change of loop intensity [KO/Control]
g
fold change of loop intensity [KO/Control]
p < 0.00663
p < 0.76
p < 0.868
rank1
rank2
rank3
rank4
fold change of the numbers of reads mapped [KO/Control]
h
fold change of expression level [KO/WT]
p < 0.280
p < 0.0184
p < 0.164
p < 0.000202
rank0
rank1
rank2
rank3
rank4
fold change of loop intensity [KO/Control]
i
fold change of expression level [KO/Control]
p < 0.000774
loop anchored
outside loops
genes classified based on location relative to loop anchors

Supplementary Figure 11. BRD9 loss dysregulates gene expression by altering chromatin loop and accessibility. (a) Comparison of the arrangement of active (red) and inactive (blue) compartments and active genomic regions mapped by H3K27ac enrichment and expression based on RNA-seq on chr1. X and y axes represent the genome coordinate and the depth of mapped read-pairs, respectively. (b) Common and specific TADs in Control and KO cells. Instances, where a single Control TAD overlapped a single KO TAD both bounded by overlapping non-TAD/boundary regions, were considered common TADs. (c) Change of loop intensities from Control to KO cells. Red and blue dots indicate Control and KO loops. X and y axes represent loop intensities detected in Control and KO cells, respectively. (d) Classification of loops based on the overlap of loop anchors between Control and KO cells: 361 Control-specific loops, one or both anchors of which do not overlap any anchor of KO loops; 2,785 KO-specific loops, one or both anchors of which do not overlap any anchor of Control loops; 548 identical loops, both anchors of which are of the same size and exactly in the same positions; 1,330 Control- and 1,333 KO-overlapped loops, anchors of which overlap on both sides but are somewhat shifted in position on the genome. (e) Comparion of the profiles of contact frequencies, BRD9, CTCF, H3K27ac, and transcription around Hgfac locus between Control and KO samples.The HiC interaction frequencies, ChIP-seq profiles of BRD9, CTCF, and H3K27ac, and RNA expression on Hgfac locus in both Control and Brd9KO are shown. The balanced HiC two-dimensional contact matrix is compared between Control (above) and KO sample (below) in top panel. The exon and intron structure of Hgfac is given in between. The color intensity represents level of interaction frequency computed at 5kb resolution. ChIP-seq and RNA-seq profiles are presented below. The positions of loop anchors are shown at the bottom. The maximum y-axis value of ChIP-seq or RNA-seq signal is set as indicated. (f) Relationship between gene expression and loop intensity for genes anchored by enhancer-promoter loops (E-P loops). A boxplot represents fold changes of the expression level of E-P loop-anchored genes for four ranks of E-P loops. A total of 1,157 combinations of genes and loops were ranked by the fold change of loop intensities from low to high in rank 1 to rank 4 (each ~ 290 combinations). Rank 0 represents a group of 2,402 non loop anchored genes, located outside of loops (of similar expression levels in Control and KO cells). Hereinafter, the exact p values computed by two-sided Mann-Whitney tests are presented, and in each boxplot, bar indicates median, box edges first and third quartile values, and whisker edges minimum and maximum values. The p values of ranks 1 to 4 were computed against rank 0. (g) Relationship between loop intensity and CTCF enrichment in loop anchors (as in Fig. 6g). The 5,024 loops were ranked by the fold change of CTCT enrichment from low to high in rank 1 to rank 4 (each ~1,256 loops). (h) Relationship between gene expression and loop intensity (as in Fig. 6i) for the same set of loops for (g). 2,151 genes expressed (FPKM ≥ 0.5) in Control or KO cells were anchored by 1,814 loops. A total of 2,573 combinations of genes and loops were ranked by the fold change of loop intensities from low to high in rank1 to rank4 (each ~ 644 combinations). (i) Difference of overall level of upregulation between loop-anchored genes and genes located outside loops. The fold changes of expression levels (KO/Control) are compared between 1,565 loop-anchored genes (left) and 2,354 genes located outside but within 100 kb of loops (right) in this boxplot. The results here are based on biological triplicate of HiC, biological duplicate of BRD9, CTCF, and H3K27ac ChIP, and biological triplicate of RNA-seq datasets for each Control and KO sample. Source data are provided as a Source Data file.

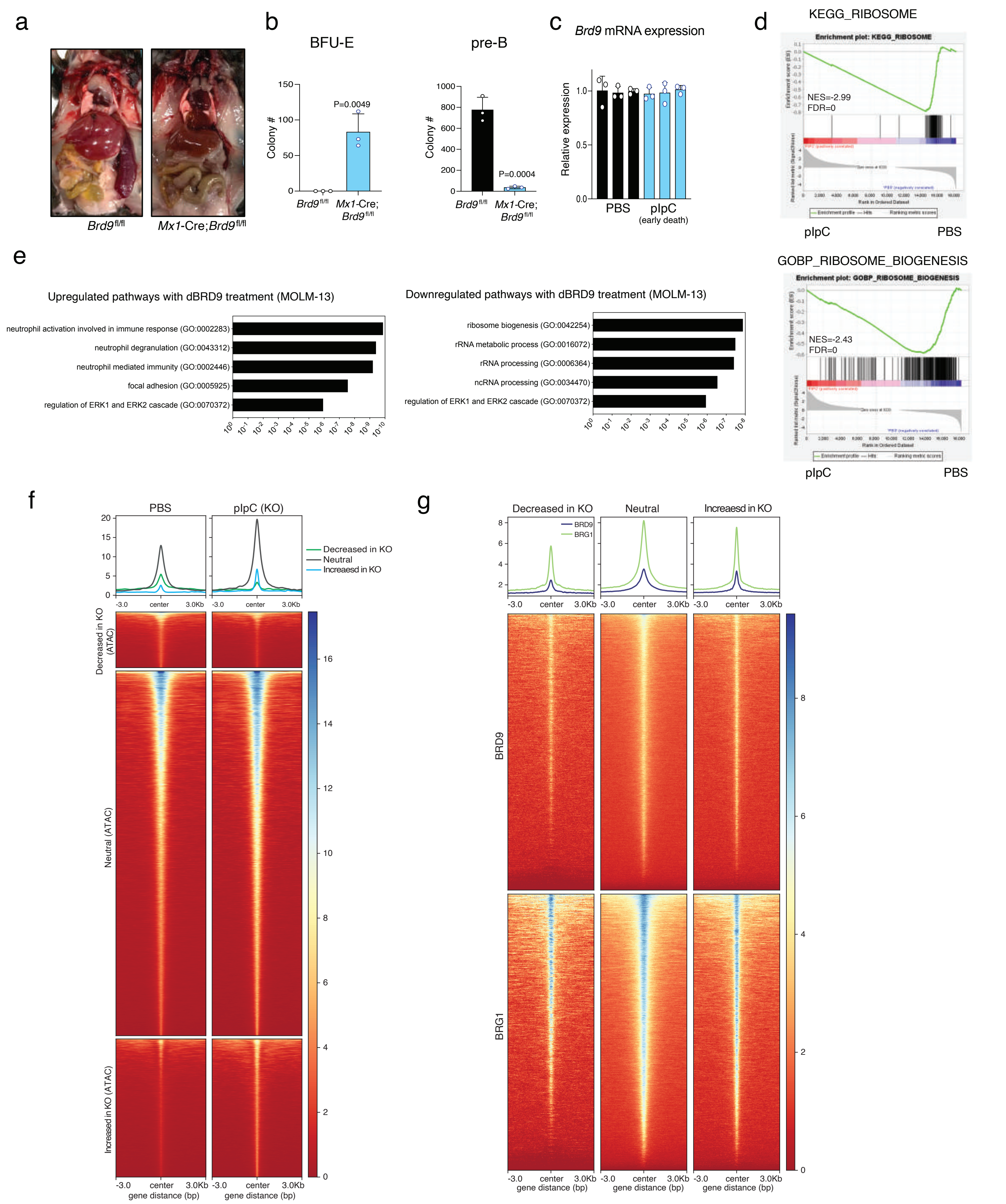

Supplementary Figure 12. BRD9 loss diminishes leukemic potential in vivo via chromatin regulation. (a) Representative hepatosplenomegaly in the Brd9fl/fl (Mx1-Cre negative) recipient of Fig. 7b. (b) Colony formation of whole BM cells. Colonies were scored 10 days after plating in methylcellulose in M3436 and M3630, for BFU-E and pre-B colonies, respectively. n = 3 independent experiments. P value relative to control by a two-sided t-test. (c) Brd9 mRNA expression of whole BM cells in mice that died within 50 days after transplant. Mean ± SD. (d) GSEA enrichment plot for ribosome-associated genes in RNA-seq of pIpC-treated group vs PBS-treated group. (e) GO terms enrichment of pathways upregulated (left) and downregulated (right) in MOLM-13 cells treated with DMSO or dBRD9 50 nM for 4 days. P values are generated via Enricher. (f) The average ATAC peaks enrichment profile over decreased peaks in pIpC (KO) (green), comparable peaks (black), and increased peaks in pIpC (KO) (blue). Heatmap illustrating the ATAC-seq signal 3 kb up and downstream. (g) The average ChIP enrichment profile of BRD9 (top) and BRG1 (bottom) over 3 groups (Decreased, Neutral, and Increased). Source data are provided as a Source Data file.

Supplementary Table 1. Numbers of read-pairs mapped, dereplicated *cis* mapped read-pairs, coverage and depth of three biological replicates.

HiC dataset	mapped read-pairs [M pairs]	Dereplicated *cis* mapped read-pairs [M pairs]	Ratio of dereplicated *cis* mapped read-pairs [%]	Coverage [%]	Depth [reads/bp]
Biological replicate 1 Control	858.1	426.8	49.7	89.0	52.9
Biological replicate 1 KO	860.6	341.1	39.6	89.0	42.3
Biological replicate 2 Control	917.4	58.4	6.4	87.1	7.4
Biological replicate 2 KO	974.5	44.3	4.5	86.4	5.6
Biological replicate 3 Control	307.7	106.3	34.5	87.9	13.3
Biological replicate 3 KO	298.9	14.3	4.8	70.1	4.2
Final merged Control	2083.2	591.4	28.4	89.0	73.1
Final merged KO	2134.0	399.7	18.7	89.0	51.0

Supplementary Table 2. Numbers of read-pairs mapped, dereplicated *cis* mapped read-pairs, coverage and depth of decreased datasets.

HiC dataset	mapped read-pairs [M pairs]	Dereplicated *cis* mapped read-pairs [M pairs]	Ratio of dereplicated *cis* mapped read-pairs	Coverage [%]	Depth [reads/bp]
3/4 of biological replicate 1 Control	569.7	318.2	55.9	88.7	39.5
3/4 of biological replicate 1 KO	566.3	271.3	47.9	88.7	33.7
1/2 of biological replicate 2 Control	379.1	230.5	60.1	88.5	28.7
1/2 of biological replicate 2 KO	375.9	207.1	55.1	88.5	25.8

Supplementary Table 3. Number and size of loops of the final and decreased datasets.

HiC dataset	Dereplicated *cis* mapped read-pairs [M pairs]	Coverage	Depth [reads/bp]	Number of loops	Size of loops [kb]
Final merged Control	28.4	89.0	73.1	2239	285.2±300.9
Final merged KO	18.7	89.0	51.0	4666	287.6±323.7
3/4 of biological replicate 1 Control	318.2	88.7	39.5	1857	267.8±260.1
3/4 of biological replicate 1 KO	271.3	88.7	33.7	4105	273.2±286.0
1/2 of biological replicate 2 Control	230.5	88.5	28.7	1509	242.0±213.7
1/2 of biological replicate 2 KO	207.1	88.5	25.8	3546	262.3±269.6

Supplementary Table 4. Number of loops and sizes at the three resolutions of the final merged datasets.

Resolution (kb)	Number of Control loops	Number of KO loops	Control loop size [kb]	KO loop size [kb]
5	149	1083	223.4±469.0	166.3±198.0
10	1162	3057	187.5±250.5	207.5±223.5
25	1737	3432	330.9±293.4	349.5±342.4
