# Supplementary material for: BRD9 determines the cell fate of hematopoietic stem cells by regulating chromatin state
Source: Nat Commun. 2023 Dec 15;14:8372. doi: 10.1038/s41467-023-44081-6 (PMC10724271; doi:10.1038/s41467-023-44081-6)
Supplement: Supplementary file 3 — Description of Additional Supplementary Files [file 41467_2023_44081_MOESM3_ESM.pdf]

Description of Additional Supplementary Files

File Name: Supplementary Data 1
Description: FACS sorting strategy for hematopoietic stem and progenitor cells (HSPCs) and mature cells in Fig. 1h.

File Name: Supplementary Data 2
Description: Transcriptome analysis of Brd9fl/fl or Mx1-Cre;Brd9fl/fl mouse BM Lin-c-Kit+cells in biological triplicate.

File Name: Supplementary Data 3
Description: Gene ontology pathway of KO versus control by RNA-seq analysis.

File Name: Supplementary Data 4
Description: Transcriptional factor motif analysis of genes upregulated in MPP3, downregulated in MPP4 and MEP of KO mice by scRNA-seq analysis. P values are indicated and generated via Enricher.

File Name: Supplementary Data 5
Description: Normalized H3K27ac and ATAC signal (RPGC) at SE and TE (detected with HOMER fdr < 0.001) in HSPCs.

File Name: Supplementary Data 6
Description: CTCF, BRG1, and BRD4 signals in control and *Brd9* KO HSPCs.

File Name: Supplementary Data 7
Description: DEGs in *Brd9* KO HSPCs.Genes whose promoter-TSS sites locate on CTCF peaks in CTCF Up, CTCF Neutral, CTCF Down and p-values of two-sided t-test were indicated.

File Name: Supplementary Data 8
Description: GO terms enrichment of the promoter-located CTCF peaks of CTCF Up group. P values are generated via Enricher.
